# Seronegative Celiac Disease and Immunoglobulin Deficiency: Where to Look in the Submerged Iceberg?

**DOI:** 10.3390/nu7095350

**Published:** 2015-09-08

**Authors:** Floriana Giorgio, Mariabeatrice Principi, Giuseppe Losurdo, Domenico Piscitelli, Andrea Iannone, Michele Barone, Annacinzia Amoruso, Enzo Ierardi, Alfredo Di Leo

**Affiliations:** 1 Section of Gastroenterology, University Hospital Policlinico, Department of Emergency and Organ Transplantation, University of Bari, 70124 Bari, Italy; E-Mails: flomic@libero.it (F.G.); b.principi@gmail.com (M.P.); giuseppelos@alice.it (G.L.); ianan@hotmail.it (A.I.); michele.barone@uniba.it (M.B.); annacinzia.amoruso@uniba.it (A.A.); ierardi.enzo@gmail.com (E.I.); 2 Section of Pathology, University Hospital Policlinico, Department of Emergency and Organ Transplantation, University of Bari, 70124 Bari, Italy; E-Mail: domenico.piscitelli@uniba.it

**Keywords:** seronegative celiac disease, tissue-transglutaminase mRNA, common variable immunodeficiency, selective IgA deficiency, selective IgM deficiency, gluten-free diet

## Abstract

In the present narrative review, we analyzed the relationship between seronegative celiac disease (SNCD) and immunoglobulin deficiencies. For this purpose, we conducted a literature search on the main medical databases. SNCD poses a diagnostic dilemma. Villous blunting, intraepithelial lymphocytes (IELs) count and gluten “challenge” are the most reliable markers. Immunohistochemistry/immunofluorescence tissue transglutaminase (tTG)-targeted mucosal immunoglobulin A (IgA) immune complexes in the intestinal mucosa of SNCD patients may be useful. In our experience, tTG-mRNA was similarly increased in seropositive celiac disease (CD) and suspected SNCD, and strongly correlated with the IELs count. This increase is found even in the IELs’ range of 15–25/100 enterocytes, suggesting that there may be a “grey zone” of gluten-related disorders. An immune deregulation (severely lacking B-cell differentiation) underlies the association of SNCD with immunoglobulin deficiencies. Therefore, CD may be linked to autoimmune disorders and immune deficits (common variable immunodeficiency (CVID)/IgA selective deficiency). CVID is a heterogeneous group of antibodies dysfunction, whose association with CD is demonstrated only by the response to a gluten-free diet (GFD). We hypothesized a familial inheritance between CD and CVID. Selective IgA deficiency, commonly associated with CD, accounts for IgA-tTG seronegativity. Selective IgM deficiency (sIgMD) is rare (<300 cases) and associated to CD in 5% of cases. We diagnosed SNCD in a patient affected by sIgMD using the tTG-mRNA assay. One-year GFD induced IgM restoration. This evidence, supporting a link between SNCD and immunoglobulin deficiencies, suggests that we should take a closer look at this association.

## 1. The Submerged Iceberg of Celiac Disease and the Dilemma of Seronegativity

Celiac disease (CD) is the most common autoimmune enteropathy. In one of the largest screening trials, a prevalence of 1:133 was calculated, meaning that about 1% of the global population is affected [1]. It is characterized by a genetic background in which human leukocyte antigens (HLA) haplotypes DQ2/DQ8 play a major role as predisposing factors [1]. An involvement of some special alleles, such as DQ A1*05 (part of the DQ2 genotype), has been invoked. Nevertheless, these haplotypes are very common in the general population, with a mean prevalence of 20%, and only a minority develops CD [1]. Therefore, the analysis of HLA haplotypes is recommended in CD diagnosis as well as in special situations, such as patients undergoing a serological/histological examination only after having started an empirical gluten-free diet. Despite these evidences, patients with diagnosed CD are much fewer than the estimated prevalence. In Italy, for instance, 600,000 people are estimated to suffer from CD, but only in 150,000 has a firm diagnosis been made [2]. This figure underlines that most affected people could belong to the “submerged iceberg” of undiagnosed CD, such as the seronegative type of disorder (SNCD), characterized by the absence of well-known serological markers (anti-endomysium antibodies (EMA) or anti-tissue transglutaminase (anti-tTG)) [3].

Moreover, the clinical spectrum of CD is extremely wide. Indeed, CD may show extra-intestinal manifestations such as iron deficiency anemia, bone loss, short stature, skin and liver disease [4]. These symptoms, in the absence of classic intestinal involvement, may delay the final diagnosis. Commonly, forms of CD characterized by predominantly extra-intestinal features are regarded as atypical CD, and they may perhaps account for the majority of cases [3,5]. Another reason that may account for the suboptimal detection rate of CD is represented by subclinical features, showing incomplete concordance among the histological, clinical and serological findings. Latent CD is a vague and largely used term indicating various conditions. Sometimes, it may denote a normal villous architecture with abnormalities such as an increased number of intraepithelial lymphocytes (IELs) and/or increased mucosal permeability even in the absence of serological markers. This condition, according to Oslo definition [3], is observed in patients on a gluten containing diet [6,7]. Potential CD is characterized by a positive CD serology and normal small intestinal mucosa [3,8], although often referred to as an increased number of IELs in the villi [9,10,11]. However, the term (potential) is used also in the case of suspected SNCD. Therefore, atypical, latent and potential CD are part of the submerged iceberg of the disorder and often are linked to SNCD. Currently, a clear indication to assume a gluten free diet does not exist for latent or potential CD.

Seronegativity is a dilemma in CD [12]. SNCD was firstly described by Abrams *et al.* [13], who evaluated the sensitivity and specificity of serology in CD patients with (Marsh 3) or without villous atrophy (Marsh 1 and 2). They found positive EMA in 77% of atrophic and only 33% of non-atrophic lesions. The study also analyzed immunoglobulin A (IgA) anti-tTG. Although only 14 subjects underwent this test, IgA anti-tTG were positive in all the patients with atrophy and absent in those with partial atrophy. Other authors later repeated this experience [14,15,16,17,18,19,20,21], as shown in Table 1, underlining that seronegativity is inversely related to the degree of villous atrophy. Epidemiological data about SNCD are scanty due to its complicated diagnostic frame, but the prevalence ranges from 1.03% among all CD patients [21] to 28% in latent CD [22].

**Table 1:** Prevalence of anti-tissue transglutaminase (anti-tTG) and endomysium antibodies (EMA) in cases of non-atrophic celiac disease (CD).

Reference	Anti-tTG Positivity	EMA Positivity
Abrams, J.A. *et al.*, 2004 [13]	0%	33%
Tursi, A. *et al.*, 2003 [14]	7.69%	Not tested
Tursi, A. *et al.*, 2003 [15]	17.1%	8.6%
Dickey, W. *et al.*, 2000 [16]	Not tested	79%
Tursi, A. *et al.*, 2001 [17]	Not tested	33%
Rostami, K. *et al.*, 1999 [18]	Not tested	31%
Kurppa, K. *et al.*, 2010 [19]	88%	Not tested
Salmi, T.T. *et al.*, 2006 [20]	28.6%	87.6% (cumulative value including atrophic CD)
Makovicky, P. *et al.*, 2013 [21]	9.1%	0%

Mucosal deposits of anti-tTG and tissue transglutaminase (tTG) may be considered the main feature of SNCD [23]. Although these deposits have been described even in individuals with overt CD, it has been shown that in SNCD, IgA anti-tTG have a great affinity for their antigen, binding strongly to tTG2, and so preventing immunocomplex deposits from being able to pass into the circulation. The strong antigen-antibody connection could explain the negative serological tests [20]. Indeed, the production of auto-antibodies in subjects with CD occurs in the intestinal mucosa, as evidenced by the presence of immune-complexes revealed by immunofluorescence. Usually, auto-antibodies cross the mucosa and enter blood vessels [24]. In SNCD, however, the antibodies may be confined in the lamina propria rather than passing into the bloodstream. Six studies [19,25,26,27,28,29] have investigated such deposits by immunofluorescence, finding amounts ranging from 64.7%–100% in 221 of 307 (72%) potential SNCD patients. Moreover, in a study by Kaukinen *et al.* [30], 41 subjects with IELs at duodenal histology were assigned to a gluten-free or gluten-containing diet, that led to a diagnosis of a gluten-related disorder in 11 of them. Deposits of immunocomplexes, detected by immunohistochemistry, were discovered in 10 of these 11 patients. Interestingly, a study [25] reported that the presence of anti-tTG deposits was a predictor of the subsequent onset of villous atrophy or histological worsening and serological positivization. In a study analyzing the affinity of anti-tTG/tTG [20], Salmi *et al.* demonstrated that after starting a gluten-free diet, the levels of deposits reduced until they were not significantly different from those in controls.

Immunoglobulin deficiencies (ID) are congenital or inherited disorders of humoral immunity characterized by low immunoglobulin titers. They could account for a part of submerged celiac iceberg, since they contribute to a lower CD detection rate, in particular for potential, latent or SNCD.

On these bases, the present narrative review was performed in order to create an overview on the link between SNCD and immunoglobulin deficiencies. For this purpose, we performed a literature search in the main medical databases (PubMed, Scopus, EMBASE and ScienceDirect), by using the following key words: celiac disease, atypical, latent, potential, seronegative, tissue transglutaminase, immunoglobulin deficiency, IgA deficiency, IgM deficiency, common variable immunodeficiency.

## 2. Immunoglobulin Deficiencies and Celiac Disease

A gastrointestinal involvement is very frequent in ID. Indeed, patients with ID show gastrointestinal symptoms in up to 50% of cases [31], and this may complicate the diagnosis of CD in primary ID. The primary function of the immune system is to protect against viruses, bacteria and non-self antigens. The fact that the gastrointestinal mucosa is the largest contact surface where this process takes place accounts for the very common gastroenterological involvement in ID. Furthermore, ID may cause microscopic alterations in the mucosa that mimic gastrointestinal disorders like CD or inflammatory bowel disease, making a correct differential diagnosis even harder.

Herein, we describe the most common relationships between ID and CD. In detail, selective IgA deficiency (sIgAD), common variable immunodeficiency (CVID) and selective IgM deficiency (sIgMD) are analyzed.

### 2.1. Selective IgA Deficiency

Selective IgA deficiency (sIgAD) is the most common primary ID, with a prevalence of 1:300–700 individuals. It is defined as serum IgA levels less than 0.07 g/L with normal IgM and IgG levels in people >4 years old [32]. The pathogenic hallmark is a defective regulation of the terminal maturation of B lymphocytes into IgA-secreting plasma cells [33]. sIgAD becomes clinically evident in childhood, because of recurrent respiratory and gastrointestinal infections. Moreover, sIgAD may frequently be associated to atopic or autoimmune disorders such as inflammatory bowel disease, nodular lymphoid hyperplasia, pernicious anemia and CD [34].

The association between CD and sIgAD may be explained by a shared genetic susceptibility. sIgAD, like CD, is strongly associated with the major histocompatibility complex (MHC) region and, in particular, with the human leukocyte antigen (HLA)-B8, DR3, DQ2 haplotype. HLA-DQ/DR is the major immunoglobulin A locus [35,36]. Up to 45% of IgAD patients have at least one copy of this haplotype, compared to 16% in the general population [37].

The prevalence of CD in sIgAD is much more than in the general population, ranging from 6.7%–20.6% as summarized in Table 2 [38,39,40,41,42,43]. On the other hand, sIgAD is more common in celiac patients, with a prevalence of 1:39 [44].

Serological tests with IgA anti-tTG are not reliable for the diagnosis of CD in sIgAD, because of the lack of IgA production. This is why IgG-tTG tests have been proposed as alternative tools for diagnostic screening [45]. Instead, the histological diagnosis may encounter some pitfalls, since the histopathology of CD in sIgAD is indistinguishable from the pathology in patients with conventional CD. A peculiar feature could be the absence of IgA-secreting plasma cells in biopsy specimens [46]. Moreover, sIgAD patients are at high risk of Giardia spp. infection, which can simulate the histopathology of CD [47]. In doubtful cases, a gluten-free diet (GFD) can be proposed, because CD in sIgAD is promptly responsive to gluten withdrawal, resulting in normalization of the atrophic lesions [48]. In this regard, Valletta, *et al.* [49] reported the case of a nine-year old girl with sIgAD and recurrent diarrhea and abdominal pain, who underwent duodenal biopsy showing partial villous atrophy. Despite seronegativity, a course of GFD was administered and yielded histological resolution of the atrophy, so the diagnosis of SNCD was hypothesized. Four years later, while she was on a gluten-containing diet, serology positivization occurred, confirming the diagnosis of CD. This report strengthens the atypical behavior of CD in ID.

**Table 2:** Main studies investigating the prevalence of celiac disease (CD) in individuals affected by selective IgA deficiency (sIgAD).

Reference	Prevalence	Test Used for Diagnosis
Bienvenu, F. *et al.*, 2014 [38]	17.7% (8/45)	IgG-tTG, IgG-DGP
Wang, N. *et al.*, 2014 [39]	12.6% (45/356)	IgG-tTG, IgG-DGP and histology
Ludvigsson, J.F. *et al.*, 2014 [40]	6.7% (167/2495)	IgG-tTG, IgG-DGP
Pituch-Noworolska, A. *et al.*, 2013 [41]	20.6% (13/63)	IgG-tTG, IgG-DGP, IgG-EMA
Lenhardt, A. *et al.*, 2004 [42]	8.7% (11/126)	IgG-tTG, IgG-DGP and histology
Korponay-Szabó, I.R. *et al.*, 2003 [43]	9.8% (17/174)	IgG-tTG, IgG-EMA
Total	8% (261/3259)	

tTG, tissue transglutaminase, DGP, deamidated gliadin peptide; EMA, endomysium antibodies.

The observation that subjects with sIgAD have a higher incidence of CD [50] has prompted some authors to look for a “pathogenetic picture” of this patient subset. Borrelli *et al.* [51] discovered that patients with both diseases had higher IEL infiltrates than those with sIgAD alone and controls. In short, an expected finding was observed, *i.e.*, a subset of IELs, the so-called γδ IELs, were over-expressed in duodenal samples of sIgAD-CD. Moreover, sIgAD-CD had more CD25+ cells (T regulatory lymphocytes) in the lamina propria than isolated sIgAD. Finally, 86% of sIgAD-CD patients showed IgM-tTG deposits, a phenomenon linked to a compensatory overproduction of IgM in response to the lack of IgA. This study represents, to the best of our knowledge, the only one which investigated immune-complexes deposits in sIgAD. This observation may suggest that mucosal immune-complexes characterize SNCD both in the presence and absence of ID. Moreover, sIgAD-CD expressed elevated levels of B-lymphocyte stimulator (BLyS), a molecule that is involved in several autoimmune diseases, while a proliferation-inducing ligand (APRIL) was significantly up-regulated only in isolated sIgAD [52]. Since APRIL promotes IgA production, its increased expression could represent a physiological negative feedback mechanism. BLyS over-expression, instead, may be invoked as a putative mechanism for the increased risk of onset of autoimmune diseases in people with sIgAD. Finally, a cytokine profile of sIgAD-CD was characterized by an enhanced production of inflammatory cytokines (namely interleukin-2, interferon gamma and tumor necrosis factor alpha), which were significantly higher than in CD or sIgAD alone, suggesting a persistent state of activation of pro-inflammatory signals in CD patients, particularly with a coexistent IgAD [53].

In conclusion, all these findings suggest that sIgAD and CD are characterized by signs of an impaired immune activation, which may account for an increased prevalence of seronegative disorder. Although several diagnostic difficulties may be encountered in the detection of SNCD in this group of patients, a strict follow-up, as well as a GFD in selected cases, could offer the best choice to gain a clearer diagnostic perspective for doubtful cases.

### 2.2. Common Variable Immunodeficiency

Common variable immunodeficiency (CVID) is a heterogeneous group of primary immunoglobulin deficiencies featuring low serum levels of immunoglobulins, a depressed response to specific antigens and high risk of recurrent infections [54]. Although the estimated prevalence is 1:25–50,000, it is considered to be the most common symptomatic ID [54]. The diagnosis relies on reduced levels of IgG and IgA, as well as on an absent or reduced antibody production in response to vaccines. Several pathogenic hypotheses have been made. A single genetic mutation has not been found, but a complex genetic background characterized by mutations and polymorphisms in genes deputed to B lymphocytes development (Inducible T-cell COStimulator: *ICOS*; B-cell activating factor receptor: *BAFF-R*; Cluster of differentiation: *CD20, CD19, CD21, CD81*) has been portrayed [55,56,57,58]. Further evidences about a possible common role played by HLA complex in both CD and CVID has been described. Indeed, several common polymorphisms in HLA loci have been detected. As reported, haplotype analysis, linkage disequilibrium, and homozygosity mapping indicated that HLA-DQ/DR is the major immunoglobulin A locus, strongly suggesting an overlapping immune pathogenesis for CVID and CD [59]. Moreover, Viallard *et al.* [60] demonstrated that CVID and CD showed altered expression of HLA-DR on antigen presenting cells (APC), thus hypothesizing that an imbalance in the process of antigen presentation by APC through HLA complex may induce an immunological response shared by CVID and CD.

CVID may show a wide range of immunological manifestations, including autoimmune phenomena, such as cytopenias, megaloblastic anemia/atrophic gastritis, immune thrombocytopenic purpura, autoimmune hemolytic anemia, sarcoidosis-like granulomatous infiltrative disorders, inflammatory bowel disease, autoimmune hepatitis and CD [61].

Gastrointestinal symptoms are very common in CVID, especially persistent diarrhea that manifests in more than 50% of cases. The corresponding histopathological pattern has been described as a sprue-like picture, resembling CD: villous atrophy and IELs infiltrate. However, unlike CD, CVID should be suspected when plasma cells are reduced or absent in the lamina propria [62,63,64].

For these reasons, patients suffering from both CD and CVID pose a clinical conundrum. It is well known that these diseases may share a common dysregulation of the ICOS molecule [65], and coexistence in the same family of patients with CD and CVID has been described [66]. Although celiac-like lesions can be observed in 30% of CVID patients, the true prevalence of CD in CVID is much lower, as reported in Table 3 [41,63,67,68,69,70], being 9.2% overall. Biagi *et al.* [70] have observed that the histologic response to a GFD is the only reliable tool to establish the diagnosis of CD in CVID. Other authors have suggested that human leukocyte antigen (HLA) determination may be helpful, since a DQ2 or DQ8 haplotype was associated with concomitant CD, whilst a “not-at-risk” haplotype in a CVID patient with villous atrophy led them to exclude CD [67]. Indeed, only patients with DQ2/8 responded with a histological and clinical improvement to a GFD and this result may be a further evidence of a link between CD and CVID, mediated by HLA haplotypes. On the other hand, CD serology has shown to be ineffective in CVID due to the high rate of false negatives, caused by the fact that CVID patients cannot mount an appropriate antibody response [71].

**Table 3:** Main studies investigating the prevalence of celiac disease (CD) in individuals affected by common variable immunodeficiency (CVID).

Reference	Prevalence
Malamut, G. *et al.*, 2010 [63]	4% (2/50)
Pituch-Noworolska, A. *et al.*, 2013 [41]	7% (3/43)
Venhoff, N. *et al.*, 2013 [67]	20% (4/20)
Rodríguez-Negrete, E.V. *et al.*, 2015 [68]	18% (3/18)
Diez, R. *et al.*, 2010 [69]	0% (0/20)
Biagi, F. *et al.*, 2012 [70]	27.3% (3/11)
Total	9.2% (15/162)

The clinicopathologic response to a GFD has been employed only in few case reports to confirm CD in CVID [72,73,74], but because of the rarity of the disease, larger prospective blinded studies are lacking, and this limits the use of GFD in this setting. Therefore, further studies involving larger sample sizes are warranted in this field.

### 2.3. Selective IgM Deficiency

Selective IgM deficiency (sIgMD) is a very rare disorder, defined by low levels of IgM—<0.40 g/L according to current guidelines [75]—in the absence of alterations of other immunoglobulin classes. In adults, the prevalence is about 1:15,000 [76].

sIgMD causes a severe alteration in maturation and function of B lymphocytes. Defects in B cell differentiation into IgM-immunoglobulin secreting cells, a reduced number of IgM-secreting B cells with a failure of secreted Igμ chain mRNA synthesis and decreased antigen proliferation IgM responses have been observed [76,77,78,79,80,81]. Confirming these alterations, Cipe *et al.* [82] found that patients with sIgMD have low levels of non-switched memory B cells.

Clinical manifestations of sIgMD include recurrent viral and bacterial infections resulting in periodic infectious dermatitis, diarrhea, meningitis, upper and lower respiratory infections and sepsis [83]. Autoimmune disorders such as hemolytic anemia, thyroiditis, rheumatoid arthritis systemic lupus erithematosus and CD have been described [83].

Gastrointestinal manifestations occur in 15.7% of patients with sIgMD, and some reports have found an association between these two disorders [84,85]. Interestingly, IgM levels returned to normal levels in most pediatric and adult patients observing a GFD. In this respect, we described the case of a sIgMD patient with SNCD [86]. The 18-year-old patient complained about abdominal pain, diarrhea and weight loss and showed villous atrophy with diffuse immature lymphocytes at duodenal biopsy, despite negative anti-tTG. However, tissue transglutaminase mRNA mucosal levels exhibited a six-fold increase. The patient was assigned to GFD and six months later the symptoms had disappeared, the villous architecture was restored and mucosal tissue transglutaminase mRNA was comparable to that of healthy subjects. A similar result (normalization of increased mucosal tissue transglutaminase mRNA) was obtained by our group in a subset of suspected SNCD patients in an ongoing study. Surprisingly, after one year of GFD, a complete restoration of normal IgM levels was seen. Moreover, we observed that the GFD caused a maturation of lymphocytes. Indeed, the mucosa was populated by numerous lymphocytes before the GFD that turned into plasma cells after starting the diet, thus explaining the increase in IgM levels.

Such secondary forms of sIgMD, occurring concomitantly to CD, could be linked to a decreased immunoglobulin synthesis by a dysfunctional lymphoreticular tissue stimulated by gluten antigen exposure [85,87].

## 3. Conclusive Remarks

The ID universe is a fascinating field in immunology due to the wide variety of clinical and autoimmune features. Gastrointestinal involvement is common and often resembles primary digestive disorders such as CD. Additionally, the lack of immunoglobulin production often accounts for the absence of serological markers of CD. For this reason, ID could mask CD, and the frequent association between CD and ID is a diagnostic challenge for the clinician, the endoscopist and the pathologist. Apart from gluten-related disorders, indeed, a condition of villous atrophy or duodenal lymphocytosis may be linked to other disorders such as alimentary atopy, inflammatory bowel disease, parasitic or viral infections and drugs such as olmesartan [22,88,89,90], all conditions demanding differential diagnosis, for which a diagnostic algorithm has been proposed [12]. In Table 4, we summarize the main causes of duodenal lymphocytosis and villous atrophy.

**Table 4:** Main causes of duodenal lymphocytosis and villous atrophy, classified according to etiological criteria.

Gluten-related	Infectious Causes	Immunological	Drugs	Other
Celiac disease	Virus (Rotavirus, Enterovirus, Adenovirus, Coronavirus)	Immunoglobulin deficiencies	Olmesartan	Irritable bowel syndrome
Gluten sensitivity	Parasites (*Giardia*)	Food allergy	Non steroidal anti-inflammatory drugs	
Seronegative celiac disease	Bacteria (*Salmonella*, *Shigella*)	Autoimmune enteritis		
Wheat allergy	Small intestinal bacterial overgrowth	Vasculitides		
	*Helicobacter pylori*	Systemic autoimmune disorders		
		Inflammatory bowel disease		
		Microscopic enteritis		

For these reasons, novel endoscopic advances have been proposed to improve the detection of CD, notably the water-immersion technique and virtual chromoendoscopy [91,92,93,94]. It is known that immunohistochemical analysis of T-cell receptor γδ (TCRγδ) [95] may be effective in discriminating CD from other causes of duodenal lymphocytosis [96,97,98,99], especially considering that an increase in IELs may be found even in non-CD conditions [100]. In this regard, Fernández-Bañares *et al.* [101] demonstrated that cytometric analysis of γδ T-cells in the duodenal mucosa displayed a specificity of 100%, much better than the 87% achieved with anti-TG2 IgA subepithelial deposit analysis. The detection of anti-tTG in the supernatant of cultured enterocytes taken from patients with SNCD has recently been proposed, and has proven to be more effective than serology. Tosco *et al.* [102] found that the measurement of antibodies secreted into culture supernatants has a higher sensitivity and specificity (97.5% and 92.3%, respectively) than the detection of mucosal deposits (77.5% and 80.0%, respectively). Moreover, in a group of 559 CD patients, Picarelli *et al.* [103] showed that anti-tTG and EMA detection in enterocyte culture improved the sensitivity and specificity by about 10% compared to traditional serology. Therefore, we highlight that analysis of the gamma-delta IELs subpopulation yields interesting results in the discrimination between gluten- and non-gluten-related enteropathies [95]. Finally, in our experience [104], tTG-mRNA was similarly increased in seropositive CD and suspected SNCD, and strongly correlated with the IEL count. This increase was found even in the IEL range of 15–25/100 enterocytes, suggesting that there may be a “grey zone” of gluten-related disorders, and that this technique could be helpful in diagnosing doubtful SNCD cases.

Finally, the genetic analysis of the HLA genes has been proposed to support the diagnosis of CD in ID. CD is known to be associated with DQ2 and DQ8 haplotypes. These haplotypes are very common in the general population, with a mean prevalence of 20%, although only a minority would develop CD [105,106]. Due to this, the analysis of HLA haplotypes is recommended [107]. The role of HLA haplotypes may also be a potential tool to recognize CD in patients with ID. Some studies confirmed that a DQ2/DQ8 positivity may be very helpful in doubtful cases [67]. Rare alleles, such as DQ A1*05, have been proven to be linked to some cases of CD (even with seronegativity) [100], but they have not been investigated in patients with ID. However, despite many attempts, a reliable tool for CD detection in ID has not yet been found. Currently, a careful histological analysis by a trained pathologist and a correct clinical study (including the response to a GFD, in particular) are the only available tools. Novel strategies, based on molecular analysis, could offer a “turning point” in this setting [12].

## References

[1] Fasano A., Berti I., Gerarduzzi T., Not T., Colletti R.B., Drago S., Elitsur Y., Green P.H., Guandalini S., Hill I.D. (2003). Prevalence of celiac disease in at-risk and not-at-risk groups in the United States: A large multicenter study. Arch. Intern. Med..

[2] Italian Celiac Disease Association. http://www.celiachia.it/AIC/AIC.aspx?SS=351.

[3] Ludvigsson J.F., Leffler D.A., Bai J.C., Biagi F., Fasano A., Green P.H., Hadjivassiliou M., Kaukinen K., Kelly C.P., Leonard J.N. (2013). The Oslo definitions for coeliac disease and related terms. Gut.

[4] Rostami Nejad M., Rostami K., Pourhoseingholi M.A., Nazemalhosseini Mojarad E., Habibi M., Dabiri H., Zali M.R. (2009). Atypical presentation is dominant and typical for coeliac disease. J. Gastrointestin. Liver Dis..

[5] Gasbarrini G., Miele L., Malandrino N., Grieco A., Addolorato G., Gasbarrini A., Gammarota G., Bonvicini F. (2009). Celiac disease in the 21st century: Issues of under- and over-diagnosis. Int. J. Immunopathol. Pharmacol..

[6] Moayyedi P., O’Mahony S., Jackson P., Lynch D.A., Dixon M.F., Axon A.T. (1997). Small intestine in lymphocytic and collagenous colitis: Mucosal morphology, permeability, and secretory immunity to gliadin. J. Clin. Pathol..

[7] Matysiak-Budnik T., Malamut G., de Serre N.P., Grosdidier E., Seguier S., Brousse N., Caillat-Zucman S., Cerf-Bensussan N., Schmitz J., Cellier C. (2007). Long-term follow-up of 61 coeliac patients diagnosed in childhood: Evolution toward latency is possible on a normal diet. Gut.

[8] Ferguson A., Arranz E., O’Mahony S. (1993). Clinical and pathological spectrum of celiac disease—Active, silent, latent, potential. Gut.

[9] Biagi F., Trotta L., Alfano C., Balduzzi D., Staffieri V., Bianchi P.I., Marchese A., Vattiato C., Zilli A., Luinetti O. (2013). Prevalence and natural history of potential celiac disease in adult patients. Scand. J. Gastroenterol..

[10] Lionetti E., Castellaneta S., Pulvirenti A., Tonutti E., Francavilla R., Fasano A., Catassi C., Italian Working Group of Weaning and Celiac Disease Risk (2012). Prevalence and natural history of potential celiac disease in at-family-risk infants prospectively investigated from birth. J. Pediatr..

[11] Auricchio R., Tosco A., Piccolo E., Galatola M., Izzo V., Maglio M., Paparo F., Troncone R., Greco L. (2014). Potential celiac children: 9-Year follow-up on a gluten-containing diet. Am. J. Gastroenterol..

[12] Ierardi E., Losurdo G., Piscitelli D., Giorgio F., Sorrentino C., Principi M., Montenegro L., Amoruso A., Di Leo A. (2015). Seronegative celiac disease: Where is the specific setting?. Gastroenterol. Hepatol. Bed Bench.

[13] Abrams J.A., Diamond B., Rotterdam H., Green P.H. (2004). Seronegative celiac disease: Increased prevalence with lesser degrees of villous atrophy. Dig. Dis. Sci..

[14] Tursi A., Brandimarte G., Giorgetti G.M. (2003). Prevalence of antitissue transglutaminase antibodies in different degrees of intestinal damage in celiac disease. J. Clin. Gastroenterol..

[15] Tursi A., Brandimarte G. (2003). The symptomatic and histologic response to a gluten-free diet in patients with borderline enteropathy. J. Clin. Gastroenterol..

[16] Dickey W., Hughes D.F., McMillan S.A. (2000). Reliance on serum endomysial antibody testing underestimates the true prevalence of coeliac disease by one fifth. Scand. J. Gastroenterol..

[17] Tursi A., Brandimarte G., Giorgetti G., Gigliobianco A., Lombardi D., Gasbarrini G. (2001). Low prevalence of antigliadin and anti-endomysium antibodies in subclinical/silent celiac disease. Am. J. Gastroenterol..

[18] Rostami K., Kerckhaert J., Tiemessen R., von Blomberg B.M., Meijer J.W., Mulder C.J. (1999). Sensitivity of antiendomysium and antigliadin antibodies in untreated celiac disease: Disappointing in clinical practice. Am. J. Gastroenterol..

[19] Kurppa K., Ashorn M., Iltanen S., Koskinen L.L., Saavalainen P., Koskinen O., Mäki M., Kaukinen K. (2010). Celiac disease without villous atrophy in children: A prospective study. J. Pediatr..

[20] Salmi T.T., Collin P., Korponay-Szabó I.R., Laurila K., Partanen J., Huhtala H., Király R., Lorand L., Reunala T., Mäki M. (2006). Endomysial antibody-negative coeliac disease: Clinical characteristics and intestinal autoantibody deposits. Gut.

[21] Makovicky P., Rimarova K., Boor A., Makovicky P., Vodicka P., Samasca G., Kruzliak P. (2013). Correlation between antibodies and histology in celiac disease: Incidence of celiac disease is higher than expected in the pediatric population. Mol. Med. Rep..

[22] DeGaetani M., Tennyson C.A., Lebwohl B., Lewis S.K., Abu Daya H., Arguelles-Grande C., Bhagat G., Green P.H. (2013). Villous atrophy and negative celiac serology: A diagnostic and therapeutic dilemma. Am. J. Gastroenterol..

[23] Gatti S., Rossi M., Alfonsi S., Mandolesi A., Cobellis G., Catassi C. (2014). Beyond the intestinal celiac mucosa: Diagnostic role of anti-TG2 deposits, a systematic review. Front. Med. (Lausanne).

[24] Korponay-Szabó I.R., Halttunen T., Szalai Z., Laurila K., Király R., Kovács J.B., Fésüs L., Mäki M. (2004). *In vivo* targeting of intestinal and extraintestinal transglutaminase 2 by coeliac autoantibodies. Gut.

[25] Tosco A., Salvati V.M., Auricchio R., Maglio M., Borrelli M., Coruzzo A., Paparo F., Boffardi M., Esposito A., D’Adamo G. (2011). Natural history of potential celiac disease in children. Clin. Gastroenterol. Hepatol..

[26] Salmi T.T., Collin P., Jarvinen O., Haimila K., Partanen J., Laurila K., Korponay-Szabo I.R., Huhtala H., Reunala T., Mäki M. (2006). Immunoglobulin A autoantibodies against transglutaminase 2 in the small intestinal mucosa predict forthcoming coeliac disease. Aliment. Pharmacol. Ther..

[27] Maglio M., Tosco A., Auricchio R., Paparo F., Colicchio B., Miele E., Rapacciuolo L., Troncone R. (2011). Intestinal deposits of anti-tissue transglutaminase IgA in childhood celiac disease. Dig. Liver Dis..

[28] Tosco A., Aitoro R., Auricchio R., Ponticelli D., Miele E., Paparo F., Greco L., Troncone R., Maglio M. (2013). Intestinal anti-tissue transglutaminase antibodies in potential coeliac disease. Clin. Exp. Immunol..

[29] Tosco A., Maglio M., Paparo F., Rapacciuolo L., Sannino A., Miele E., Barone M.V., Auricchio R., Troncone R. (2008). Immunoglobulin A anti-tissue transglutaminase antibody deposits in the small intestinal mucosa of children with no villous atrophy. J. Pediatr. Gastroenterol. Nutr..

[30] Kaukinen K., Peräaho M., Collin P., Partanen J., Woolley N., Kaartinen T., Nuutinen T., Halttunen T., Mäki M., Korponay-Szabo I. (2005). Small-bowel mucosal transglutaminase 2-specific IgA deposits in coeliac disease without villous atrophy: A prospective and randomized clinical study. Scand. J. Gastroenterol..

[31] Agarwal S., Mayer L. (2013). Diagnosis and treatment of gastrointestinal disorders in patients with primary immunodeficiency. Clin. Gastroenterol. Hepatol..

[32] Notarangelo L.D., Fischer A., Geha R.S., Casanova J.L., Chapel H., Conley M.E., Cunningham-Rundles C., Etzioni A., Hammartröm L., International Union of Immunological Societies Expert Committee on Primary Immunodeficiencies (2009). Primary immunodeficiencies: 2009 Update. J. Allergy Clin. Immunol..

[33] Conley M.E., Cooper M.D. (1981). Immature IgA B cells in IgA-deficient patients. N. Engl. J. Med..

[34] Brandtzaeg P. (2010). Update on mucosal immunoglobulin A in gastrointestinal disease. Curr. Opin. Gastroenterol..

[35] Cunningham-Rundles C., Fotino M., Rosina O., Peter J.B. (1991). Selective IgA deficiency, IgG subclass deficiency, and the major histocompatibility complex. Clin. Immunol. Immunopathol..

[36] Olerup O., Smith C.I., Hammarström L. (1990). Different amino acids at position 57 of the HLA-DQ beta chain associated with susceptibility and resistance to IgA deficiency. Nature.

[37] Mohammadi J., Ramanujam R., Jarefors S., Rezaei N., Aghamohammadi A., Gregersen P.K., Hammarström L. (2010). IgA deficiency and the MHC: Assessment of relative risk and microheterogeneity within the HLA A1 B8, DR3 (8.1) haplotype. J. Clin. Immunol..

[38] Bienvenu F., Anghel S.I., Besson Duvanel C., Guillemaud J., Garnier L., Renosi F., Lachaux A., Bienvenu J. (2014). Early diagnosis of celiac disease in IgA deficient children: Contribution of a point-of-care test. BMC Gastroenterol..

[39] Wang N., Truedsson L., Elvin K., Andersson B.A., Rönnelid J., Mincheva-Nilsson L., Lindkvist A., Ludvigsson J.F., Hammarström L., Dahle C. (2014). Serological assessment for celiac disease in IgA deficient adults. PLoS ONE.

[40] Ludvigsson J.F., Neovius M., Hammarström L. (2014). Association between IgA deficiency and other autoimmune conditions: A population-based matched cohort study. J. Clin. Immunol..

[41] Pituch-Noworolska A., Błaut-Szlósarczyk A., Zwonarz K. (2013). Occurrence of autoantibodies for gastrointestinal autoimmune diseases in children with common variable immune deficiency and selected IgA deficiency. Prz. Gastroenterol..

[42] Lenhardt A., Plebani A., Marchetti F., Gerarduzzi T., Not T., Meini A., Villanacci V., Martelossi S., Ventura A. (2004). Role of human-tissue transglutaminase IgG and anti-gliadin IgG antibodies in the diagnosis of coeliac disease in patients with selective immunoglobulin A deficiency. Dig. Liver Dis..

[43] Korponay-Szabó I.R., Dahlbom I., Laurila K., Koskinen S., Woolley N., Partanen J., Kovács J.B., Mäki M., Hansson T. (2003). Elevation of IgG antibodies against tissue transglutaminase as a diagnostic tool for coeliac disease in selective IgA deficiency. Gut.

[44] Wang N., Shen N., Vyse T.J., Anand V., Gunnarson I., Sturfelt G., Rantapää-Dahlqvist S., Elvin K., Truedsson L., Andersson B.A. (2011). Selective IgA deficiency in autoimmune diseases. Mol. Med..

[45] Villalta D., Tonutti E., Prause C., Koletzko S., Uhlig H.H., Vermeersch P., Bossuyt X., Stern M., Laass M.W., Ellis J.H. (2010). IgG antibodies against deamidated gliadin peptides for diagnosis of celiac disease in patients with IgA deficiency. Clin. Chem..

[46] Villalta D., Alessio M.G., Tampoia M., Tonutti E., Brusca I., Bagnasco M., Pesce G., Bizzaro N. (2007). Diagnostic accuracy of IgA anti-tissue transglutaminase antibody assays in celiac disease patients with selective IgA deficiency. Ann. N. Y. Acad. Sci..

[47] Eren M., Saltik-Temizel I.N., Yüce A., Cağlar M., Koçak N. (2007). Duodenal appearance of giardiasis in a child with selective immunoglobulin A deficiency. Pediatr. Int..

[48] Klemola T. (1988). Immunohistochemical findings in the intestine of IgA-deficient persons: Number of intraepithelial T lymphocytes is increased. J. Pediatr. Gastroenterol. Nutr..

[49] Valletta E., Fornaro M., Pecori S., Zanoni G. (2011). Selective immunoglobulin A deficiency and celiac disease: Let’s give serology a chance. J. Investig. Allergol. Clin. Immunol..

[50] Cataldo F., Marino V., Ventura A., Bottaro G., Corazza G.R. (1998). Prevalence and clinical features of selective immunoglobulin A deficiency in coeliac disease: An Italian multicentre study. Italian Society of Paediatric Gastroenterology and Hepatology (SIGEP) and “Club del Tenue” Working Groups on Coeliac Disease. Gut.

[51] Borrelli M., Maglio M., Agnese M., Paparo F., Gentile S., Colicchio B., Tosco A., Auricchio R., Troncone R. (2010). High density of intraepithelial gammadelta lymphocytes and deposits of immunoglobulin (Ig)M anti-tissue transglutaminase antibodies in the jejunum of coeliac patients with IgA deficiency. Clin. Exp. Immunol..

[52] Fabris M., De Vita S., Visentini D., Fabro C., Picierno A., Lerussi A., Villalta D., Alessio M.G., Tampoia M., Tonutti E. (2009). B-lymphocyte stimulator and a proliferation-inducing ligand serum levels in IgA-deficient patients with and without celiac disease. Ann. N. Y. Acad. Sci..

[53] Cataldo F., Lio D., Marino V., Scola L., Crivello A., Corazza G.R. (2003). Plasma cytokine profiles in patients with celiac disease and selective IgA deficiency. Pediatr. Allergy Immunol..

[54] Abolhassani H., Sagvand B.T., Shokuhfar T., Mirminachi B., Rezaei N., Aghamohammadi A. (2013). A review on guidelines for management and treatment of common variable immunodeficiency. Expert. Rev. Clin. Immunol..

[55] Grimbacher B., Hutloff A., Schlesier M., Glocker E., Warnatz K., Dräger R., Eibel H., Fischer B., Schäffer A.A., Mages H.W. (2003). Homozygous loss of ICOS is associated with adult-onset common variable immunodeficiency. Nat. Immunol..

[56] Vences-Catalán F., Kuo C.C., Sagi Y., Chen H., Kela-Madar N., van Zelm M.C., van Dongen J.J., Levy S. (2015). A mutation in the human tetraspanin CD81 gene is expressed as a truncated protein but does not enable CD19 maturation and cell surface expression. J. Clin. Immunol..

[57] Lougaris V., Gallizzi R., Vitali M., Baronio M., Salpietro A., Bergbreiter A., Salzer U., Badolato R., Plebani A. (2012). A novel compound heterozygous TACI mutation in an autosomal recessive common variable immunodeficiency (CVID) family. Hum. Immunol..

[58] Van de Ven A.A., Compeer E.B., Bloem A.C., van de Corput L., van Gijn M., van Montfrans J.M., Boes M. (2012). Defective calcium signaling and disrupted CD20-B-cell receptor dissociation in patients with common variable immunodeficiency disorders. J. Allergy Clin. Immunol..

[59] Kralovicova J., Hammarström L., Plebani A., Webster A.D., Vorechovsky I. (2003). Fine-scale mapping at IGAD1 and genome-wide genetic linkage analysis implicate HLA-DQ/DR as a major susceptibility locus in selective IgA deficiency and common variable immunodeficiency. J. Immunol..

[60] Viallard J.F., Blanco P., André M., Etienne G., Liferman F., Neau D., Vidal E., Moreau J.F., Pellegrin J.L. (2006). CD8+HLA-DR+ T lymphocytes are increased in common variable immunodeficiency patients with impaired memory B-cell differentiation. Clin. Immunol..

[61] Cunningham-Rundles C. (2008). Autoimmune manifestations in common variable immunodeficiency. J. Clin. Immunol..

[62] Lougaris V., Ravelli A., Villanacci V., Salemme M., Soresina A., Fuoti M., Lanzarotto F., Lanzini A., Plebani A., Bassotti G. (2015). Gastrointestinal Pathologic Abnormalities in Pediatric- and Adult-Onset Common Variable Immunodeficiency. Dig. Dis. Sci..

[63] Malamut G., Verkarre V., Suarez F., Viallard J.F., Lascaux A.S., Cosnes J., Bouhnik Y., Lambotte O., Béchade D., Ziol M. (2010). The enteropathy associated with common variable immunodeficiency: The delineated frontiers with celiac disease. Am. J. Gastroenterol..

[64] Luzi G., Zullo A., Iebba F., Finaldi V., Sanchez Mete L., Muscaritoli M., Aiuti F. (2003). Duodenal pathology and clinical-immunological implications in common variable immunodeficiency patients. Am. J. Gastroenterol..

[65] Haimila K., Einarsdottir E., de Kauwe A., Koskinen L.L., Pan-Hammarström Q., Kaartinen T., Kurppa K., Ziberna F., Not T., Vatta S. (2009). The shared CTLA4-ICOS risk locus in celiac disease, IgA deficiency and common variable immunodeficiency. Genes Immun..

[66] Licinio R., Principi M., Amoruso A., Piscitelli D., Ierardi E., Di Leo A. (2013). Celiac disease and common variable immunodeficiency: A familial inheritance?. J. Gastrointestin. Liver Dis..

[67] Venhoff N., Emmerich F., Neagu M., Salzer U., Koehn C., Driever S., Kreisel W., Rizzi M., Effelsberg N.M., Kollert F. (2013). The role of HLA DQ2 and DQ8 in dissecting celiac-like disease in common variable immunodeficiency. J. Clin. Immunol..

[68] Rodríguez-Negrete E.V., Mayoral-Zavala A., Rodríguez-Mireles K.A., Díaz de León-Salazar O.E., Hernández-Mondragón O., Gómez-Jiménez L.M., Moreno-Alcántar R., González-Virla B. (2015). Prevalence of gastrointestinal disorders in adults with common variable immunodeficiency at Specialty Hospital Dr. Bernardo Sepulveda. Rev. Alerg. Mex..

[69] Díez R., García M.J., Vivas S., Arias L., Rascarachi G., Pozo E.D., Vaquero L.M., Miguel A., Sierra M., Calleja S. (2010). Gastrointestinal manifestations in patients with primary immunodeficiencies causing antibody deficiency. Gastroenterol. Hepatol..

[70] Biagi F., Bianchi P.I., Zilli A., Marchese A., Luinetti O., Lougaris V., Plebani A., Villanacci V., Corazza G.R. (2012). The significance of duodenal mucosal atrophy in patients with common variable immunodeficiency: A clinical and histopathologic study. Am. J. Clin. Pathol..

[71] Daniels J.A., Lederman H.M., Maitra A., Montgomery E.A. (2007). Gastrointestinal tract pathology in patients with common variable immunodeficiency (CVID): A clinicopathologic study and review. Am. J. Surg. Pathol..

[72] Cecinato P., Fuccio L., Sabattini E., Laterza L., Caponi A., Azzaroli F., Mazzella G. (2014). An unusual cause of weight loss in a young Caucasian man. Common variable immunodeficiency (CVI) associated with diffuse enteral nodular lymphoid hyperplasia (NLH) and CD. Gut.

[73] Béchade D., Desramé J., De Fuentès G., Camparo P., Raynaud J.J., Algayres J.P. (2004). Common variable immunodeficiency and celiac disease. Gastroenterol. Clin. Biol..

[74] Bili H., Nizou C., Nizou J.Y., Coutant G., Schmoor P., Algayres J.P., Daly J.P. (1997). Common variable immunodeficiency and total villous atrophy regressive after gluten-free diet. Rev. Med. Interne.

[75] Dati F., Schumann G., Thomas L., Aguzzi F., Baudner S., Bienvenu J., Blaabjerg O., Blirup-Jensen S., Carlström A., Petersen P.H. (1996). Consensus of a group of professional societies and diagnostic companies on guidelines for interim reference ranges for 14 proteins in serum based on the standardization against the IFCC/BCR/CAP Reference Material (CRM 470). International Federation of Clinical Chemistry. Community Bureau of Reference of the Commission of the European Communities. College of American Pathologists. Eur. J. Clin. Chem. Clin. Biochem..

[76] Ohno T., Inaba M., Kuribayashi K., Masuda T., Kanoh T., Uchino H. (1987). Selective IgM deficiency in adults: Phenotypically and functionally altered profiles of peripheral blood lymphocytes. Clin. Exp. Immunol..

[77] Inoue T., Okumura Y., Shirama M., Ishibashi H., Kashiwagi S., Okubo H. (1986). Selective partial IgM deficiency: Functional assessment of T and B lymphocytes *in vitro*. J. Clin. Immunol..

[78] Kimura S., Tanigawa M., Nakahashi Y., Inoue M., Yamamura Y., Kato H., Sugino S., Kondo M. (1993). Selective IgM deficiency in a patient with Hashimoto’s disease. Intern. Med..

[79] Karsh J., Watts C.S., Osterland C.K. (1982). Selective immunoglobulin M deficiency in an adult: Assessment of immunoglobulin production by peripheral blood lymphocytes *in vitro*. Clin. Immunol. Immunopathol..

[80] Kondo N., Ozawa T., Kato Y., Motoyoshi F., Kasahara K., Kameyama T., Orii T. (1992). Reduced secreted μ mRNA synthesis in selective IgM deficiency of Bloom’s syndrome. Clin. Exp. Immunol..

[81] Yamasaki T. (1992). Selective IgM deficiency: Functional assessment of peripheral blood lymphocytes *in vitro*. Intern. Med..

[82] Cipe F.E., Doğu F., Güloğlu D., Aytekin C., Plat M., Biyikli Z., Ikincioğullari A. (2013). B-cell subsets in patients with transient hypogammaglobulinemia of infancy, partial IgA deficiency, and selective IgM deficiency. J. Investig. Allergol. Clin. Immunol..

[83] Goldstein M.F., Goldstein A.L., Dunsky E.H., Dvorin D.J., Belecanech G.A., Shamir K. (2008). Pediatric selective IgM immunodeficiency. Clin. Dev. Immunol..

[84] Hobbs J.R., Hepner G.W. (1968). Deficiency of γM-globulin in coeliac disease. Lancet.

[85] Brown D.L., Cooper A.G., Hepner G.W. (1969). IgM metabolism in coeliac disease. Lancet.

[86] Montenegro L., Piscitelli D., Giorgio F., Covelli C., Fiore M.G., Losurdo G., Iannone A., Ierardi E., Di Leo A., Principi M. (2014). Reversal of IgM deficiency following a gluten-free diet in seronegative celiac disease. World J. Gastroenterol..

[87] Blecher T.E., Brzechwa-Ajdukiewicz A., McCarthy C.F., Read A.E. (1969). Serum immunoglobulins and lymphocyte transformation studies in coeliac disease. Gut.

[88] Carroccio A., D’Alcamo A., Mansueto P. (2015). Nonceliac wheat sensitivity in the context of multiple food hypersensitivity: New data from confocal endomicroscopy. Gastroenterology.

[89] Koot B.G., Ten Kate F.J., Juffrie M., Rosalina I., Taminiau J.J., Benninga M.A. (2009). Does *Giardia lamblia* cause villous atrophy in children: A retrospective cohort study of the histological abnormalities in giardiasis. J. Pediatr. Gastroenterol. Nutr..

[90] Ianiro G., Bibbò S., Montalto M., Ricci R., Gasbarrini A., Cammarota G. (2014). Systematic review: Sprue-like enteropathy associated with olmesartan. Aliment. Pharmacol. Ther..

[91] Ianiro G., Gasbarrini A., Cammarota G. (2013). Endoscopic tools for the diagnosis and evaluation of celiac disease. World J. Gastroenterol..

[92] Cammarota G., Ianiro G., Sparano L., La Mura R., Ricci R., Larocca L.M., Landolfi R., Gasbarrini A. (2013). Image-enhanced endoscopy with I-scan technology for the evaluation of duodenal villous patterns. Dig. Dis. Sci..

[93] Cammarota G., Cazzato A., Genovese O., Pantanella A., Ianiro G., Giorgio V., Montalto M., Vecchio F.M., Larocca L.M., Gasbarrini G. (2009). Water-immersion technique during standard upper endoscopy may be useful to drive the biopsy sampling of duodenal mucosa in children with celiac disease. J. Pediatr. Gastroenterol. Nutr..

[94] Singh R., Nind G., Tucker G., Nguyen N., Holloway R., Bate J., Shetti M., George B., Tam W. (2010). Narrow-band imaging in the evaluation of villous morphology: A feasibility study assessing a simplified classification and observer agreement. Endoscopy.

[95] Lonardi S., Villanacci V., Lorenzi L., Lanzini A., Lanzarotto F., Carabellese N., Volta U., Facchetti F. (2013). Anti-TCR gamma antibody in celiac disease: The value of count on formalin-fixed paraffin-embedded biopsies. Virchows Arch..

[96] Rostami K., Aldulaimi D., Holmes G., Johnson M.W., Robert M., Srivastava A., Fléjou J.F., Sanders D.S., Volta U., Derakhshan M.H. (2015). Microscopic enteritis: Bucharest consensus. World J. Gastroenterol..

[97] Shmidt E., Smyrk T.C., Faubion W.A., Oxentenko A.S. (2013). Duodenal intraepithelial lymphocytosis with normal villous architecture in pediatric patients: Mayo Clinic experience, 2000–2009. J. Pediatr. Gastroenterol. Nutr..

[98] Aziz I., Key T., Goodwin J.G., Sanders D.S. (2015). Predictors for Celiac Disease in Adult Cases of Duodenal Intraepithelial Lymphocytosis. J. Clin. Gastroenterol..

[99] Rosinach M., Esteve M., González C., Temiño R., Mariné M., Monzón H., Sainz E., Loras C., Espinós J.C., Forné M. (2012). Lymphocytic duodenosis: Aetiology and long-term response to specific treatment. Dig. Liver Dis..

[100] Losurdo G., Piscitelli D., Giangaspero A., Principi M., Buffelli F., Giorgio F., Montenegro L., Sorrentino C., Amoruso A., Ierardi E. (2015). Evolution of nonspecific duodenal lymphocytosis over 2 years of follow-up. World J. Gastroenterol..

[101] Fernández-Bañares F., Carrasco A., García-Puig R., Rosinach M., González C., Alsina M., Loras C., Salas A., Viver J.M., Esteve M. (2014). Intestinal intraepithelial lymphocyte cytometric pattern is more accurate than subepithelial deposits of anti-tissue transglutaminase IgA for the diagnosis of celiac disease in lymphocytic enteritis. PLoS ONE.

[102] Tosco A., Auricchio R., Aitoro R., Ponticelli D., Primario M., Miele E., Rotondi Aufiero V., Discepolo V., Greco L., Troncone R. (2014). Intestinal titres of anti-tissue transglutaminase 2 antibodies correlate positively with mucosal damage degree and inversely with gluten-free diet duration in coeliac disease. Clin. Exp. Immunol..

[103] Picarelli A., Di Tola M., Marino M., Libanori V., Borghini R., Salvi E., Donato G., Vitolo D., Tiberti A., Marcheggiano A. (2013). Usefulness of the organ culture system when villous height/crypt depth ratio, intraepithelial lymphocyte count, or serum antibody tests are not diagnostic for celiac disease. Transl. Res..

[104] Ierardi E., Amoruso A., Giorgio F., Principi M., Losurdo G., Piscitelli D., Buffelli F., Fiore M.G., Mongelli A., Castellaneta N.M. (2015). Mucosal molecular pattern of tissue transglutaminase and interferon gamma in suspected seronegative celiac disease at Marsh 1 and 0 stages. Saudi J. Gastroenterol..

[105] Alarida K., Harown J., di Pierro M.R., Drago S., Catassi C. (2010). HLA-DQ2 and -DQ8 genotypes in celiac and healthy Libyan children. Dig. Liver Dis..

[106] Liu E., Lee H.S., Aronsson C.A., Hagopian W.A., Koletzko S., Rewers M.J., Eisenbarth G.S., Bingley P.J., Bonifacio E., Simell V. (2014). Risk of pediatric celiac disease according to HLA haplotype and country. N. Engl. J. Med..

[107] Rubio-Tapia A., Hill I.D., Kelly C.P., Calderwood A.H., Murray J.A. (2013). ACG clinical guidelines: Diagnosis and management of celiac disease. Am. J. Gastroenterol..

